# CD103-positive CSC exosome promotes EMT of clear cell renal cell carcinoma: role of remote MiR-19b-3p

**DOI:** 10.1186/s12943-019-0997-z

**Published:** 2019-04-11

**Authors:** Lu Wang, Guang Yang, Danfeng Zhao, Jiaqi Wang, Yang Bai, Qiang Peng, Hongzhi Wang, Ruizhe Fang, Guang Chen, Zhichao Wang, Keliang Wang, Guangbin Li, Yinhui Yang, Ziqi Wang, Pengyu Guo, Li Peng, Dayong Hou, Wanhai Xu

**Affiliations:** 1 Department of Urology (Heilongjiang Key Laboratory of Scientific Research in Urology), the Fourth Hospital of Harbin Medical University, No. 37 Yi-Yuan Street, Nangang District, Harbin, Heilongjiang Province 150081 People’s Republic of China; 2 Department of Neurosurgery, The First Affiliated Hospital of Harbin Medical University, Harbin, Heilongjiang Province People’s Republic of China

**Keywords:** Cancer stem cells, miR-19b-3p, Epithelial-mesenchymal transition, CD103^+^ exosomes, Lung metastasis

## Abstract

**Background:**

Clear cell renal cell carcinoma (CCRCC) is characterized by a highly metastatic potential. The stromal communication between stem cells and cancer cells critically influences metastatic dissemination of cancer cells.

**Methods:**

The effect of exosomes isolated from cancer stem cells (CSCs) of CCRCC patients on the progress of epithelial-mesenchymal transition (EMT) and lung metastasis of CCRCC cells were examined.

**Results:**

CSCs exosomes promoted proliferation of CCRCC cells and accelerated the progress of EMT. Bioactive miR-19b-3p transmitted to cancer cells by CSC exosomes induced EMT via repressing the expression of PTEN. CSCs exosomes derived from CCRCC patients with lung metastasis produced the strongest promoting effect on EMT. Notably, CD103^+^ CSC exosomes were enriched in tumor cells and in lung as well, highlighting the organotropism conferred by CD103. In addition, CD103^+^ exosomes were increased in blood samples from CCRCC patients with lung metastasis.

**Conclusions:**

CSC exosomes transported miR-19b-3p into CCRCC cells and initiated EMT promoting metastasis. CD103^+^ acted to guide CSC exosomes to target cancer cells and organs, conferring the higher metastatic capacity of CCRCC to lungs, suggesting CD103^+^ exosomes as a potential metastatic diagnostic biomarker.

**Graphical abstract:**

ᅟ
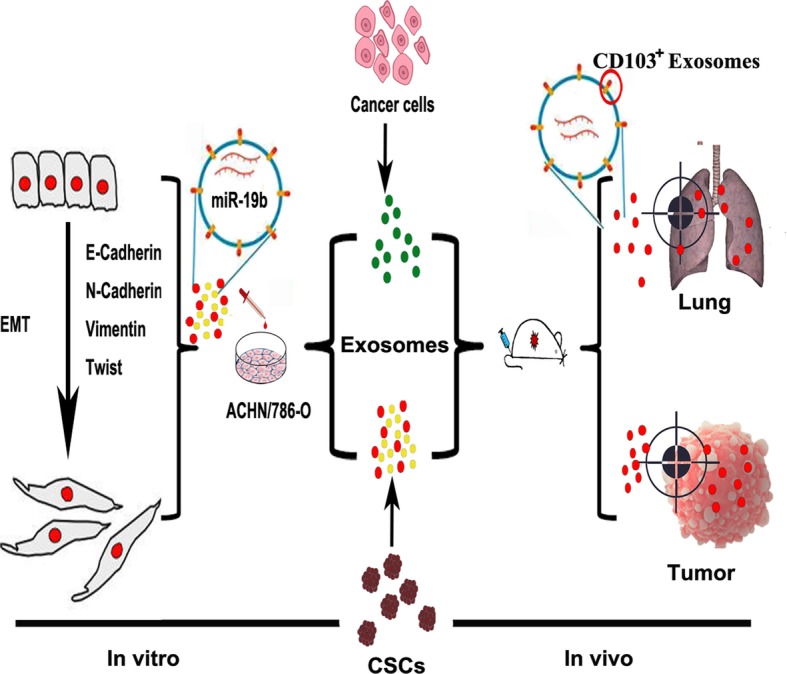

**Electronic supplementary material:**

The online version of this article (10.1186/s12943-019-0997-z) contains supplementary material, which is available to authorized users.

## Background

Renal carcinoma is among the 10 most common cancers, representing 3.7% of all new cancer cases. The incidence has been kept rising worldwide and the prognosis remains poor. Clear cell renal cell carcinoma (CCRCC), a renal cortical tumor characterized by malignant epithelial cells, is the most common type of renal carcinomas, constituting 80% of all cases. Over 30% of patients present with metastasis at the time of diagnosis [1–3]. Cancer stem cell (CSC) is a critical factor during CCRCC development because it promotes metastatic spread [4]. CCRCC stem cells (CCRCC-CSCs) express marker gene CD105 and stem cell markers Nestin, Nanog, and Oct-3/4. Despite successful isolation of CSCs in 2008, insufficient progress has been made towards decoding the roles of CSCs governing CCRCC development in the past decade [5]. It is of paramount importance to understand the molecular mechanisms by which CSCs mediate and accelerate CCRCC metastasis to develop efficient strategies for suppressing metastasis. The study reported by Grange et al. demonstrated that exosomes from CCRCC-CSCs accelerate the metastasis of cancer cells in tumor xenografts mice [6]. If CSCs derived exosomes is an indispensable approach for distant metastases in CCRCC.

Exosomes (30–150 nm in diameter) can be secreted from multiple types of cells and they participate in intercellular communications by transmitting intracellular cargoes (such as proteins and nucleic acids) into sensitive target cells [7, 8]. Exosomes have gained considerable attention in patients with urogenital cancers, both as mediators of intercellular signaling and as potential sources for the discovery of novel cancer biomarkers [9]. Exosomes released into tumor microenvironment and bloodstream known as ubiquitous messengers, have a strong impact to distant tissues [10]. Exosomes help cancer cells take root in other organs far from primary tumor site, or metastasize, the process that ultimately leads to the overwhelming majority of cancer-associated deaths (about 90%) [11, 12]. Exosomes derived from gastric cancer [13] and breast cancer [14] can initiate the pre-metastatic niche in the liver, lung and brain. Specially, stem cell-exosomes in premetastatic niche formation and biological characteristics of the cancerous stromal elements may offer novel targeting opportunities and have prognostic and predictive values [15]. Epithelial-mesenchymal transition (EMT) and mesenchymal-epithelial transition (MET) trigger the dissociation of carcinoma cells from primary carcinomas, which subsequently migrate and disseminate to distant sites. As Nicholas Syn and David Lyden introduced EMT/MET is an essential progress of exosome-mediated metastasis [16–18]. Owning to the important contribution of EMT to specific stages during metastatic progression [19], the regulatory effects of exosomes on metastasis may actually be mediated by their regulation on EMT.

The substances or molecules (such as microRNAs or miRNAs) incorporated [20] in exosomes and the transmittal destination [21] of exosomes co-determine their cellular functions. MiRNAs are small noncoding RNAs (18–24 nucleotides) that direct post-transcriptional repression of complementary mRNAs via binding 3′-UTR of miRNAs, initiating a powerful regulatory mechanism controlling a variety of cellular functions in development and metastasis of cancer [22]. The miRNA incorporated in exosomes can be transported to the pre-metastatic niche enabling wide spread influence on the gene expression of target cells. The exosomes transfer of PTEN-targeting miRNAs to metastatic tumour cells, which increase brain metastasis [23]. Exosomal transfer of miR-21 confers paclitaxel resistance in ovarian cancer cells [24]. The metastatic organotropism is one of the cancer’s greatest mysteries. Some findings raise the question of how exosomes are directed to the specific metastatic organs enabling organotropic metastatic growth [20]. Exosomes released by melanoma cells prepare sentinel lymph nodes for tumor metastasis [25]; exosomes from breast cancer cells trend to locate to lung in orthotropic nude-mouse models [26]; exosomes secreted from gastric cancer cells can be delivered into the liver [13]. The question is why exosomes preferably metastasize to certain organs? Hoshino et al. have revealed that tumour exosome integrins determine organotropic metastasis. Lung-tropic exosomes expressed integrins α6, β4 and β1; liver-tropic exosomes expressed integrins β5 and αv; brain-tropic exosomes expressed integrins β3 and αv [21, 27]. Emerging evidence supports the notion that exosomal cargo including miRNA regulates development and metastasis of cancer cells. However, the roles of exosomes from CSCs are poorly understood.

We proposed based upon the messages presented above that CSC exosomes play an important role in determining the destination or target organ/tissue for distant metastasis of CCRCC by actin of miRNAs for delivery of epigenetic information. The present study was designed to examine our hypothesis with the following specific objectives: to explore the possible role of CSC exosomes from CCRCC patients without metastasis or with lung-metastasis in the development of CCRCC, to discriminate the relative contributions of lung-metastatic and non-metastatic CSC exosomes, elucidate the signaling and cellular/molecular mechanisms underlying the effects of CSC exosomes with a particular focus on a miRNA miR-19b-3p and EMT for remote regulatory properties of CSC exosomes.

## Methods

### Human tissue and blood samples

All clinical samples were collected with written informed consent from patients in the First/Third/Forth Affiliated Hospital of Harbin Medical University, and the ethical approval was granted by the Committees for Ethical Review of Harbin Medical University. Blood and original tumor specimens from 133 CCRCC patients at stages I and II (located CCRCC) and from 76 patients at stages III and IV (metastatic CCRCC) who underwent surgery. The collected tissues were immediately divided into two parts used for primary cultures and snap-frozen in liquid nitrogen, respectively. This study was approved by the Institutional Review Board of Harbin Medical University, and all subjects provided their informed consent.

### Patient-derived CCRCC cells and cancer stem cells (CSCs)

CSCs were identified and isolated from human renal carcinomas as previously described [5]. Briefly, each CCRCC tissue specimen was minced into 1 mm^3^ cube chunks and enzymatically dissociated to single cells. Cells were isolated, using anti-CD105 antibody coupled to magnetic beads. CD105^+^ cells were maintained in CCRCC stem cell culture medium. CD105^−^ renal tumor cells were plated and maintained in DMEM with 10% exosome-depleted FBS. Finally, the cells were placed in an incubator at 37 °C with 5% CO_2_ and saturated humidity.

### Cell culture

The human renal clear cell carcinoma (CCRCC) ACHN and 786-O cells, and human embryonic kidney (HEK) 293 cells were obtained from the American Type Culture Collection (ATCC). 786-O cells were maintained in RPMI-1640 Medium. ACHN and HEK293 were maintained in DMEM medium. The medium was supplemented with 10% fetal bovine serum (Invitrogen, USA).

### Exosomes isolation and application

Exosomes were isolated as described by Liu et al [12]. Briefly, patient-derived cells were starved for 12 h (without FBS), after which the medium was collected, centrifuged at 300 g for 10 min, and 20,000 g for 20 min at 4 °C to remove cellular debris. Next, the supernatant was filtered using a 0.2 mm filter and centrifuged at 100,000×g for 90 min at 4 °C. The final pellet containing exosomes was re-suspended in PBS and the amount and size of exosomes were determined by NanoSight.

Protein content of exosomes was quantified using BCA Protein Assay. For in vitro application of exosomes, 5 × 10^5^ cells were incubated with 50 μg exosomes, and for in vivo application of exosomes, 5 mg exosomes were intravenously injected into BALB/c nude mice (SLAC Laboratory Animal Company, China) via tail vein.

### Transmission electron microscopy

Selected samples were fixed in 2.5% glutaraldehyde in 0.1 mol/L phosphate-buffered saline (PBS; pH 7.4) and fixed at 4 °C overnight. The specimens were then rinsed in buffer, post-fixed in PBS 1% OsO_4_ for 1–2 h. The samples were embedded in 10% gelatin and fixed in glutaraldehyde at 4 °C. They were then cut into blocks, stained en bloc in uranyl acetate, dehydrated in ethanol, and embedded in epoxy resin by standard procedures. The ultra-thin sections were electron-stained and observed under an electron microscope (JEM-1220, JEOL Ltd., Japan).

### Wound healing assay

CCRCC cells were seeded in six-well plates in culture medium, and grown to 70% confluence. They were then rinsed with phosphate-buffered saline (PBS). A sterile 200 μL pipette tip was used to create wounds, and the cells were incubated with exosomes for 48 h before the assessment of cell migration across the wound line.

### Invasion assay

CCRCC cells (1 × 10^5^) were seeded into upper chambers. The chambers were then inserted into transwell apparatus (Costar, USA). The upper chambers were coated with Matrigel (BD Biosciences, USA) when cell invasion assay was done. Medium with 10% FBS was added to the lower chamber. After 48 h, cells on the bottom of the inserts were fixed in 4% paraformaldehyde and stained with 0.05% crystal violet. Then cells that invaded into the lower surface were counted. Each experiment was repeated at least three times.

### RNA extraction and quantitative real-time polymerase chain reaction

Total RNA was extracted and purified using a miRNeasy Mini Kit (Qiagen, USA). Quantitative real-time polymerase chain reaction (qRT-PCR) was performed in triplicate in the ABI 7500 fast real-time PCR System (Applied Biosystems, USA). The relative expression level of miR-19b-3p was calculated through normalization to U6 internal controls, and mRNAs were normalized with Actin. The following primers were used for PCR detection:

miR-19b-3p:

5′-GTGCAAATCCATGCAAAACTGA-3′(F),

5′-GTGCAGGGTCCGAGGTGCT-3′ (R)

E-cadherin:

5′-AGAACGCATTGCCACATACACTC -3′ (F),

5′-CATTCTGATCGGTTACCGTGATC -3′ (R)

N-cadherin:

5′-ACAGTGGCCACCTACAAAGG -3′ (F),

5′-CCGAGATGGGGTTGATAATG -3′ (R)

Vimentin:

5′-GAGAACTTTGCCGTTGAAGC -3′ (F),

5′-GCTTCCTGTAGGTGGCAATC -3′ (R)

Twist:

5′- GGAGTCCGCAGTCTTACGAG -3′ (F),

5′-TCTGGAGGACCTGGTAGAGG -3′ (R)

### Protein extraction and western blot

Exosomes or cells were lysed with RIPA buffer containing a complete protease inhibitor tablet (Roche, Switzerland). Lysate was separated by 10% sodium dodecyl sulfate polyacrylamide gel electrophoresis, and the gel was blotted onto polyvinylidene fluoride (PVDF) membrane (Millipore, USA). The membrane was blocked in 5% nonfatmilk, and then incubated with either rabbit anti-human E-cadherin (3195, Cell Signaling Technologies, USA), N-cadherin (14,215, Cell Signaling Technologies, USA), Vimentin (5741, Cell Signaling Technologies), Twist (ab49254, Abcam, USA), PTEN (ab32199, Abcam, USA), CD103 (GTX64393, Genetex, USA), CD9 (20597–1-AP; Proteintech, USA), or Actin (3700, Cell Signaling Technology, USA). After washing, the membrane was incubated with the fluorescence-conjugated anti-mouse or anti-rabbit IgG (Invitrogen, USA). The bound secondary antibody was quantified using the Odyssey v1.2 software (LI-COR, USA) by measuring the band intensity (area × optical density) for each group and then normalized with Actin. The final results are expressed as fold changes by normalizing the data to control values.

### Animal studies

CCRCC cells (1 × 10^6^) suspension was subcutaneously injected into the flank of 5-week-old female athymic BALB/c nude mice (SLAC Laboratory Animal Company, China). Meanwhile, a dosage of 5 mg exosomes administered into mice via tail vein injection once every 3 days for 2 weeks. Growth rate of tumors was determined by measuring tumor size at fixed time points as to be specified. Tumor size was measured with calipers after the tumor cell injection once every 7 days for a total period of 4 weeks. Tumor volume was determined using the formula: volume = length × width^2^/2.

To evaluate metastasis, cells (stable luciferase transfected CCRCC cells) were injected into nude mice through tail vein. Tumors derived from stable luciferase-transfected cells were imaged to observe luciferase expression on day 28 after tumor cell injection. Briefly, the animals were anesthetized and then injected (i.p.) with luciferin at 150 mg/kg in a volume of 100 μL. Images were captured at a peak time of 15–20 min after injection using an IVIS-200 Imaging System (Xenogen Corporation, USA).

For in vivo exosome-tracking experiments, purified exosomes were fluorescently labeled with PKH26 membrane dye (Sigma, USA). Labeled exosomes (1 mg) were intravenously injected into mice. Organs were collected at 12 h after exosome injection and analyzed using Living Image software (Xenogen Corporation, USA).

All animal experiments were undertaken in accordance with the NIH Guide for the Care and Use of Laboratory Animals, with the approval of the Institutional Animal Care and Use Committee of Harbin Medical University.

### Statistical analysis

Statistical analysis was performed with SPSS13.0 software. Student t test, ANOVA, or chi-square analysis was applied, where appropriate. Survival rates were estimated using the Kaplan-Meier method, and survival curves were compared using the log-rank test. A probability of < 0.05 (*) or < 0.01 (**) was considered significant.

## Results

### The isolation and identification CSCs exosomes derived from CCRCC patients

**Fig. 1:**
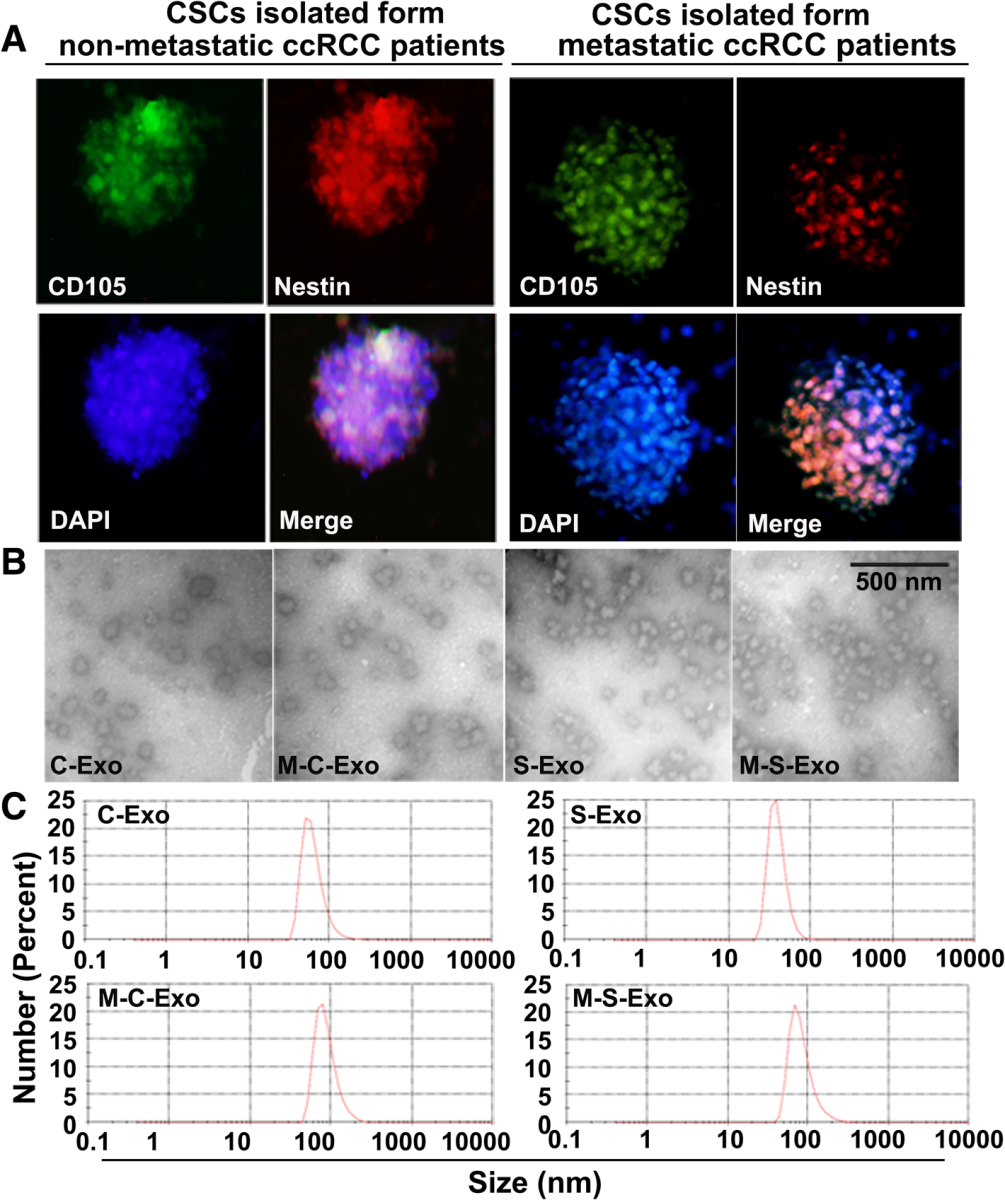
Characterization and measuring of exosomes isolated from CCRCC patients. **a** Immunofluorescent image showing the positive expression of CCRCC stem cells (CSCs) markers CD105 and Nestin. **b** Representative transmission electron micrographic images of four types of exosomes from CCRCC patients of different pathological stages and cell origins. C-Exo: exosomes isolated from cancer cells of non-metastatic CCRCC patients; M-C-Exo: exosomes isolated from cancer cells of lung-metastatic CCRCC patients; S-Exo: exosomes isolated from cancer stem cells (CSCs) of non-metastatic CCRCC patients; M-C-Exo: exosomes isolated from CSCs of lung-metastatic CCRCC patients. **c** Nanoparticle tracking showing the diameter of the particles

Tumor tissues were collected from CCRCC patients undergoing surgery without metastasis or with lung-metastasis, and primary cell culture was conducted with isolated tumor cells. CCRCC stem cell-specific marker CD105 and Nestin were stained positive in cultured CSCs (Fig. 1a). Cancer cell exosomes and CSC exosomes were then isolated from medium of non-metastatic (C-Exo and S-Exo) and metastatic CCRCC patients (M-C-Exo and M-S-Exo), respectively. Transmission electron microscopic examination showed morphological manifestations of exosomes (Fig. 1b). Exosomes were isolated, and then the size and concentration of the exosomes were measured using nanoparticle tracking analysis. The diameter of the exosome was in the size range of the exosome (Fig. 1c).

### CSC exosomes accelerate EMT of CCRCC cells

**Fig. 2:**
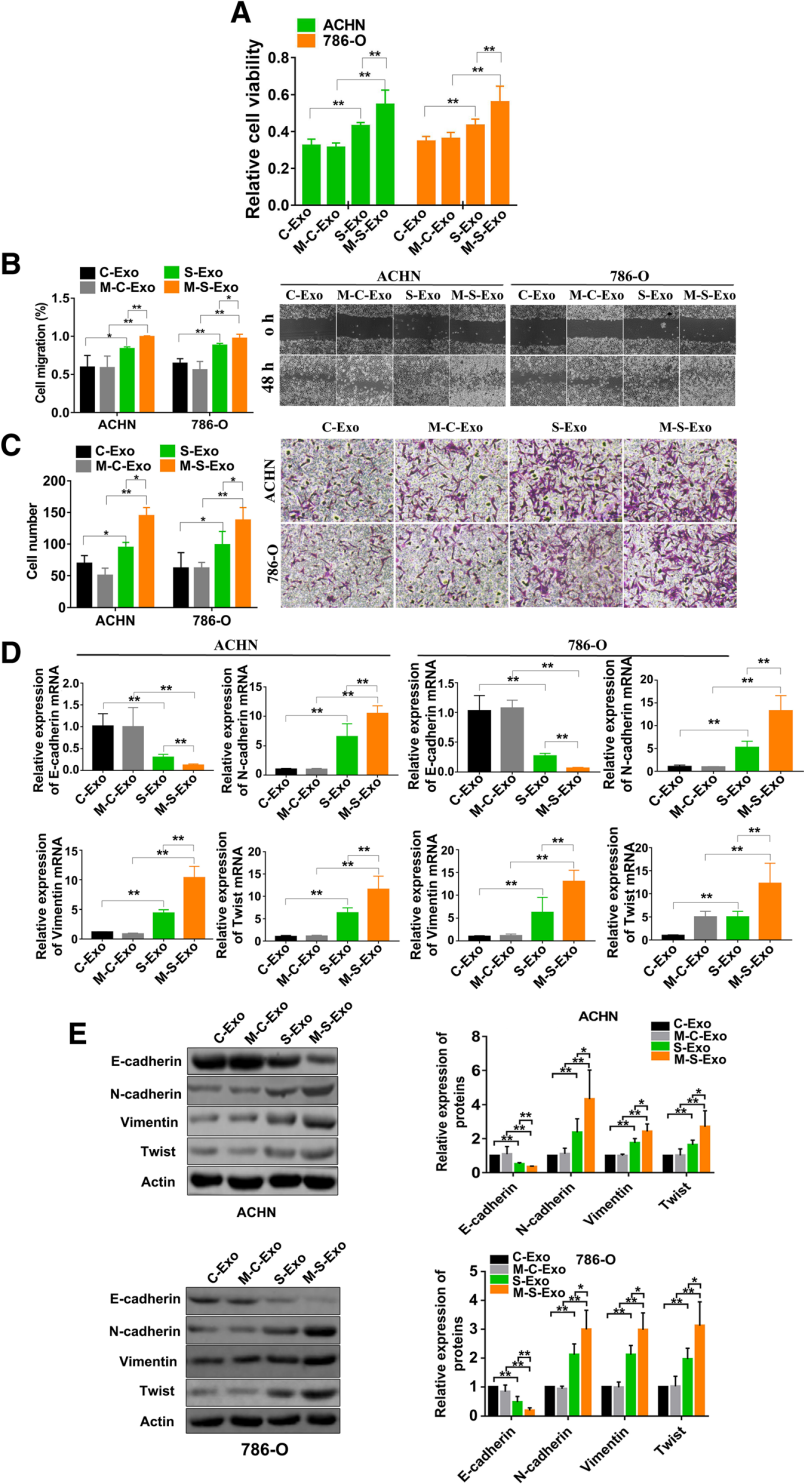
CSCs exosomes accelerate EMT progress of CCRCC cells. **a** CSCs exosomes induced cell viability of ACHN and 786-O cells. Cell number was counted via MTT assay after adding exosomes. *n* = 10 for ACHN and 786-O, **P < 0.01 relative to the controls. **b** CSCs exosomes promoted migration of ACHN and 786-O cells as reported by wound healing assay. Photomicrographs at 200× magnification. *n* = 4, *P < 0.05, **P < 0.01 relative to the controls. **c** CSCs exosomes promoted invasion of ACHN and 786-O cells as revealed by transwell assay. *n* = 4, *P < 0.05, **P < 0.01 relative to the controls. **d** The expression levels of four EMT-related genes (E-cadherin, N-cadherin, Vimentin, and Twist) in ACHN and 786-O cells were quantified using qRT-PCR. *n* = 5 for ACHN and 786-O, **P < 0.01 relative to the controls. **e** The protein levels of four EMT-related genes after treating with varying exosomes determined by western blot. *n* = 5 for ACHN and 786-O, *P < 0.05, **P < 0.01 relative to the controls. *n* = 5 for ACHN; *n* = 5 for E-cadherin, Vimentin and Twist and *n* = 6 for N-cadherin in 786-O, *P < 0.05, **P < 0.01 relative to the controls

To characterize the cellular function of CSCs and cancer cell exosomes, a number of assays were performed in CCRCC cell lines ACHN and 786-O. As illustrated in Fig. 2a, the viability of ACHN and 786-O cells were significantly increased after addition of CSC exosomes isolated either from non-metastatic (S-Exo) or metastatic (M-S-Exo) CCRCC patients. Wound healing and transwell assay further demonstrated that both S-Exo and M-S-Exo considerably accelerated the migration and invasion of ACHN and 786-O cells with substantially greater efficacies than cancer exosomes (Fig. 2b & c). Additionally, M-S-Exo elicited significantly stronger effects on viability, migration and invasion of ACHN and 786-O cells than S-Exo.

It is known that epithelial cell can acquire its migratory capacity through epithelial-mesenchymal transition (EMT) that plays a crucial role in initiating cancer metastasis. To explore whether EMT is also involved in the promoting effects of CSC exosomes on migration and invasion, we quantified the changes of mRNA and protein levels of EMT biomarkers N-cadherin, Vimentin and Twist. As anticipated, S-Exo and M-S-Exo both decreased the levels of E-cadherin relative to C-Exo and M-C-Exo (Fig. 2d & e). In sharp contrast, S-Exo and M-S-Exo both significantly upregulated the expression of N-cadherin, Vimentin and Twist in both mRNA (Fig. 2d) and protein (Fig. 2e) levels. It was noted that M-S-Exo elicited greater effects than S-Exo on the expression of EMT-related genes.

### The effects of CSC exosomes are mediated by miR-19b-3p transportation

**Fig. 3:**
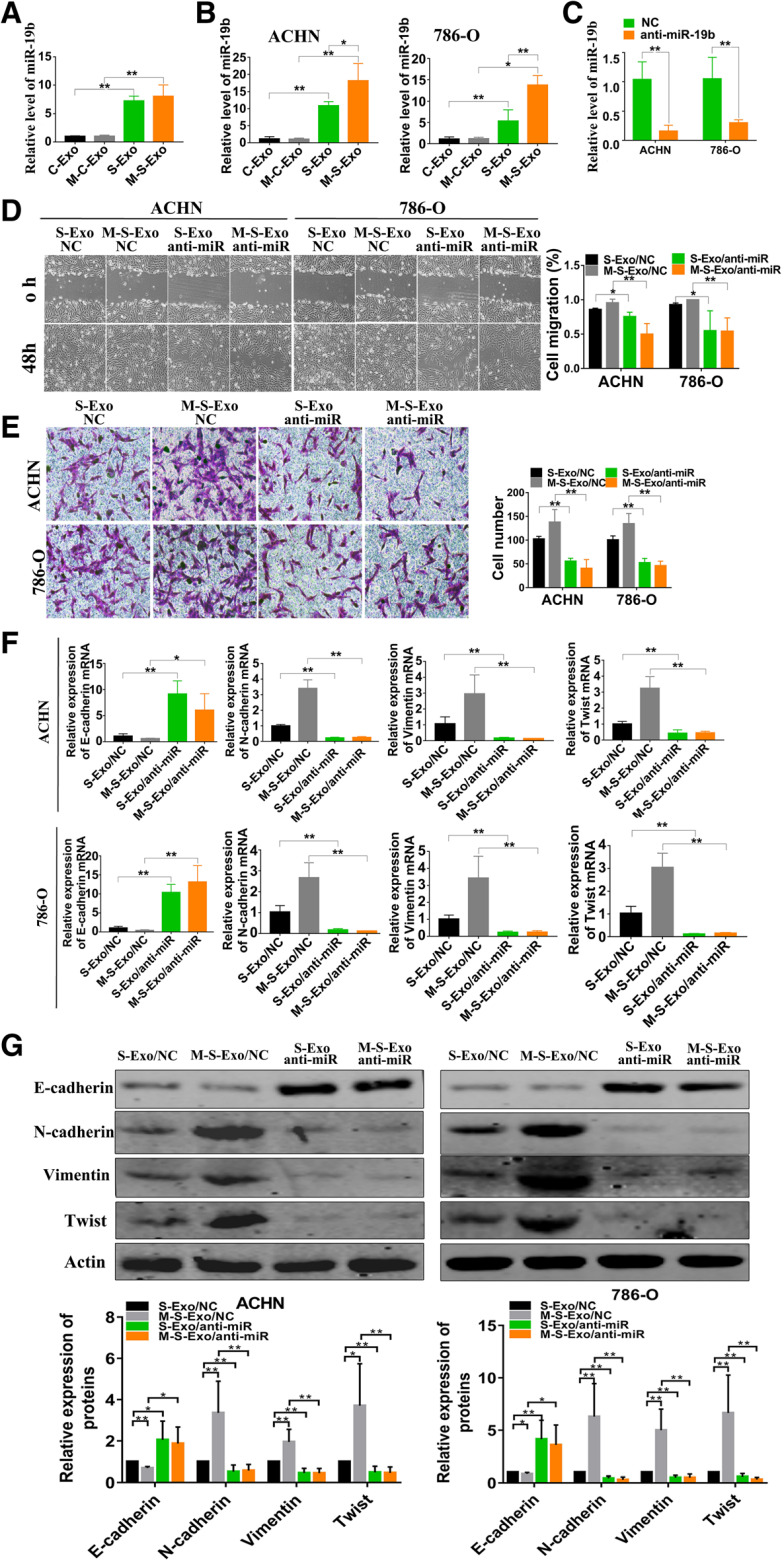
CSCs exosomes induced effects is mediated by miR-19b-3p transportation. **a** qRT-PCR analysis of relative contents of miR-19b-3p in C-Exo, M-C-Exo, S-Exo and M-S-Exo. *n* = 5, **P < 0.01 relative to the controls. **b** The expression levels of miR-19b-3p in ACHN and 786-O cells after treating with four different exosomes for 48 h. *n* = 5, *P < 0.05, **P < 0.01 relative to the controls. **c** Endogenous miR-19b-3p of CSCs were knock down by lentivirus, then the levels of miR-19b-3p loaded in CSCs exosomes were detected. *n* = 5 for ACHN and 786-O, **P < 0.01 relative to the controls. **d** The results of wound healing assay showing the effects of knockdown of endogenous miR-19b-3p by it inhibitor incorporated into lentivirus vector (anti-miR) on migration of ACHN and 786-O cells pretreated with four different exosomes for 48 h. NC represents the negative control construct for anti-miR (same below). *n* = 4, *P < 0.05, **P < 0.01 relative to the controls. **e** The results of transwell assay demonstrating the effects of anti-miR on invasion of ACHN and 786-O cells treated with four different exosomes for 48 h. *n* = 5, **P < 0.01 relative to the controls. **f** The effects of anti-miR on the expression of EMT-related genes E-cadherin, N-cadherin, Vimentin and Twist mRNAs in ACHN and 786-O cells after treating with four different exosomes for 48 h. *n* = 5, *P < 0.05, **P < 0.01 relative to the controls. **g** The effects of anti-miR on the protein levels of E-cadherin, N-cadherin, Vimentin and Twist mRNAs in ACHN and 786-O cells after treating with four different exosomes for 48 h. *n* = 5 for ACHN and *n* = 5 for E-cadherin, Vimentinand Twist and *n* = 6 for N-cadherin in 786-O, *P < 0.05, **P < 0.01 relative to the controls. *n* = 5 for ACHN; *n* = 6 for N-cadherin, Vimentin and Twist, and *n* = 5 for E-cadherin in 786-O, *P < 0.05, **P < 0.01 relative to the controls

Recent studies suggest that exosomes can transport some bioactive molecules (such as miRNAs) contained by them to the distant organs/tissues and these molecules then can fulfill their cellular functions remotely; such a mechanism is believed to play a significant role in the microenvironment for tumor development [24]. This message prompted us to exploit if miRNAs incorporated in CSC exosomes mediate the malignant effects of CSCs in canerogenesis. To this end, we focused our study on miR-19b-3p, for it has been documented to be differentially expressed between CSC exosomes and cancer exosomes in a published study [6]. We first verified the differential expression of miR-19b-3p between CSC and cancer exosomes using qRT-PCR. Our results showed that the levels of miR-19b-3p in CSC exosomes were significantly higher than in cancer exosomes, and were nearly identical between C-Exo and M-C-Exo and between S-Exo and M-S-Exo (Fig. 3a). Notably, the ACHN and 786-O cells treated with CSC exosomes from either non-metastatic or metastatic CCRCC tissues had remarkably elevated miR-19b-3p levels, and such an elevation was significantly greater after treatment with CSC exosomes from M-S-Exo than from S-Exo (Fig. 3b).

We then subsequently looked at the effects of alterations of miR-19b-3p packaged in exosomes. The lentivirus carrying the antisense inhibitor of miR-19b (anti-miR) was used to knock down endogenous miR-19b-3p and the levels of miR-19b-3p in CSCs exosomes were successfully decreased by lentivirus infection of the CSCs (Fig. 3c). As depicted in Fig. 3d & e, infection of the cells with anti-miR to knock down endogenous miR-19b-3p attenuated the migration- and invasion-promoting effects of S-Exo and M-S-Exo. Likewise, the upregulation of E-cadherin, N-cadherin, vimentin and Twist expression induced by S-Exo and M-S-Exo was also reversed by anti-miR (Fig. 3f & g). In all the cases, the negative control constructs of anti-miR (NC) tended to produce the opposite effects to anti-miR (Fig. 3d-g).

### MiR-19b-3p promotes EMT via targeting PTEN

**Fig. 4:**
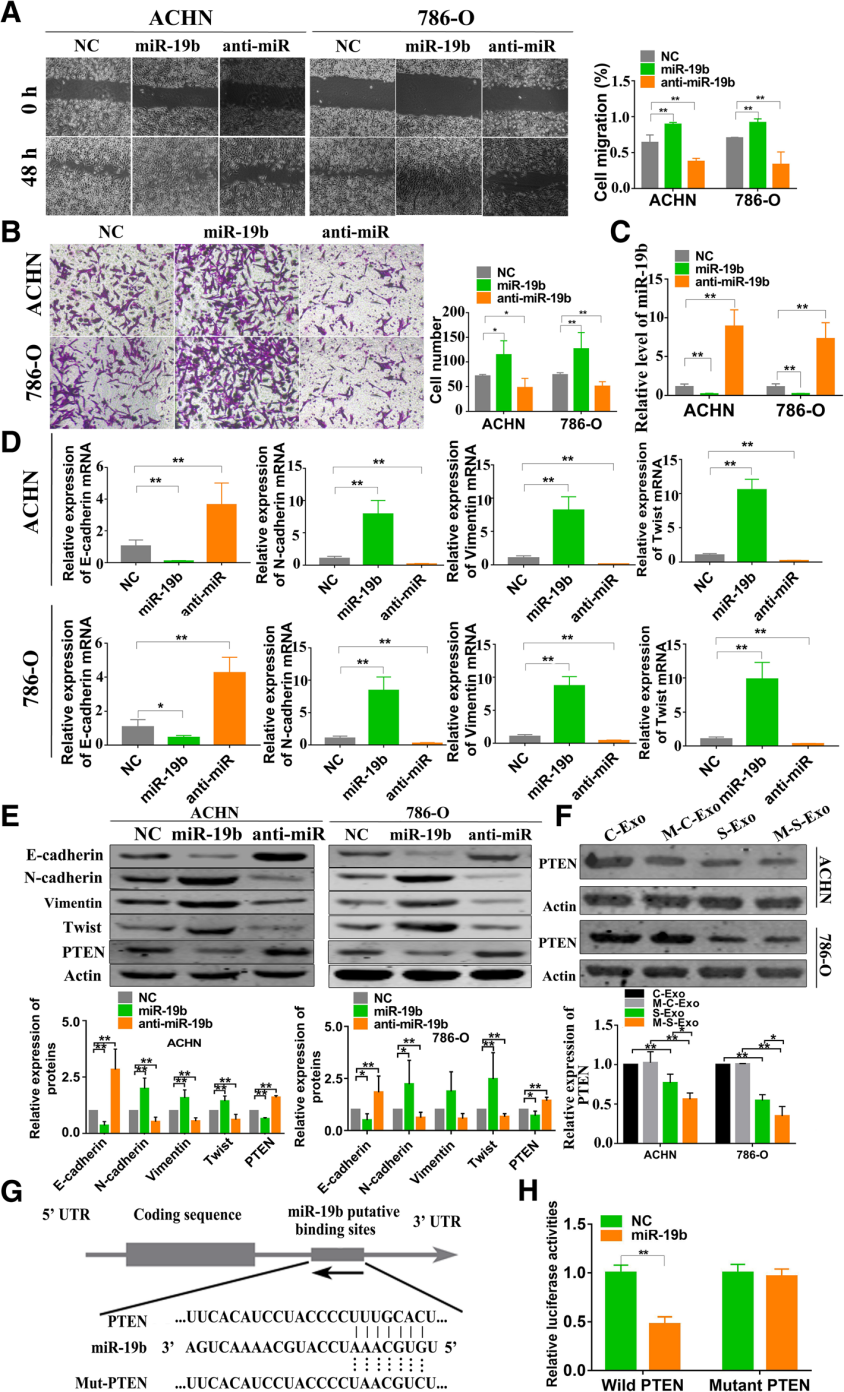
MiR-19b-3p promotes EMT via targeting PTEN. **a** The results of wound healing assay showing the effects of infected miR-19b-3p or miR-19b-3p inhibitor (anti-miR) lentivirus on migration of ACHN and 786-O cells respectively. *n* = 4, **P < 0.01 relative to the controls. NC represents the negative control (same below). **b** The results of transwell assay showing the effects of miR-19b-3p or anti-miR on invasion of ACHN and 786-O cells. *n* = 5, **P < 0.01 relative to the controls. **c** miR-19b-3p expression was quantified using qRT-PCR. *n* = 5, **P < 0.01 relative to the controls. **d** qRT-PCR results showing the effects of miR-19b-3p or anti-miR on the expression of E-cadherin, N-cadherin, Vimentin, and Twist in ACHN and 786-O cells. *n* = 5, **P < 0.01 relative to the controls. **e** Western blots results showing the effects of miR-19b-3p or anti-miR on protein levels of E-cadherin, N-cadherin, Vimentin and Twist in ACHN and 786-O cells. *n* = 6 for E-cadherin, Vimentin and Twist and *n* = 5 for N-cadherin in ACHN; *n* = 7 for E-cadherin, N-cadherin and Twist and *n* = 6 for Vimentin in 786-O. *P < 0.05, **P < 0.01 relative to the controls. **f** Changes of protein levels of PTEN after treatment with varying exosomes in ACHN and 786-O cells. All experiments were repeated at least 3 times with duplicate samples, *P < 0.05; **P < 0.001. **g** Sequence alignment between PTEN mRNA and miR-19b-3p showing the matched nucleotides in the seed site of miR-19b-3p and the nucleotide-substitution mutation of the seed site of PTEN. **h** Luciferase reporter gene assay showing the inhibitory effects of miR-19b-3p mimic on luciferase activities in HEK293 cells. NC: The negative control construct of miR-19b-3p mimic. From B to H, *n* = 5 for all data, *P < 0.05, relative to the control

The above results indicate that miR-19b-3p might be an initiator of EMT since knockdown of this miRNA resulted in downregulation of N-cadherin, Vimentin and Twist. If this is true, then miR-19b-3p should impose positive effects on migration and invasion and on expression of these EMT-related genes. Indeed, infection ACHN and 786-O cells with miR-19b-3p lentivirus in enhanced migration and invasion just as CSC exosomes did (Fig. 4a & b). MiR-19b-3p was successfully regulated by infection as showed in Fig. 4c. Meanwhile, miR-19b-3p markedly up-regulated the expression of N-cadherin, Vimentin and Twist in both mRNA and protein levels, and down-regulated E-cadherin (Fig. 4d & e).

To decipher the target mechanisms underlying the effects of miR-19b-3p, we performed computational analysis with the TargetScan and miRBase databases, and identified PTEN as a potential target for miR-19b-3p. Aberrant expression of PTEN has been implicated as a key mediator of cell migration which is closely related to EMT [23, 28, 29]. We therefore went on to investigate the regulatory effect of miR-19b-3p on PTEN. Our results demonstrated that the protein level of PTEN in CCRCC cells infected with miR-19b-3p lentivirus was robustly down-regulated relative to the cells transfected with the negative control construct (Fig. 4e). Consistently, S-Exo or M-S-Exo also decreased the expression of PTEN compared with cancer exosomes in ACHN and 786-O cells (Fig. 4f).

To assess whether there is a direct interaction between miR-19b-3p and PTEN gene, we subcloned the 3′-UTR of PTEN mRNA into the Dual-luciferase reporter plasmid system (Fig. 4g). Subsequently, miR-19b-3p mimic or negative control construct (NC) was co-transfected with the luciferase plasmid into HEK293 cells and luciferase activities were then determined. We observed considerable decrease in luciferase activity in cells transfected with miR-19b-3p mimic. However, when nucleotide-substitution mutations were introduced to the predicted binding site of miR-19b-3p in the 3′-UTR of PTEN mRNA, miR-19b-3p lost its ability to affect luciferase activity (Fig. 4h). The negative control construct of miR-19b-3p mimic (NC) failed to influence the expression of EMT biomarkers, PTEN expression, and luciferase activities.

### CSC exosomes accelerate tumorigenesis and metastasis of CCRCC cells in vivo

**Fig. 5:**
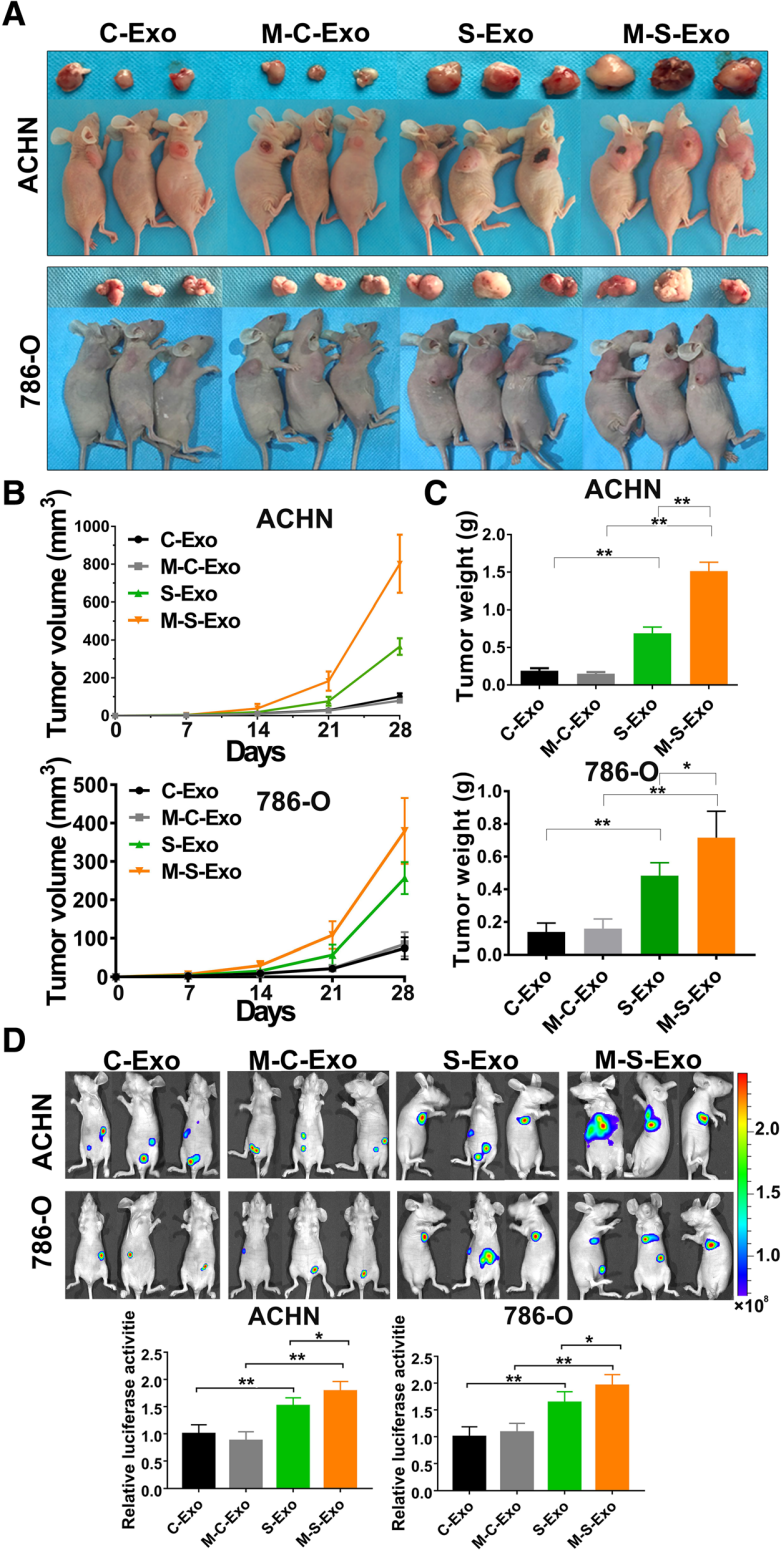
CSCs exosomes promote tumorigenesis and metastasis of CCRCC cells in vivo. **a** Representative tumors excised from xenografts in nude mice implanted with CCRCC cells for 4 weeks. Note that S-Exo and M-S-Exo produced greater promoting effects on tumors than C-Exo and M-C-Exo, and the malignant effects of M-S-Exo was stronger than S-Exo. **b** Growth of human tumor xenografts in nude mice. **c** Excised tumor weight on day 28. **d** 4 weeks following injection of tumor cells, mice were injected intraperitoneally with luciferin and imaged using a Xenogen IVIS imaging system. From A to D, *n* = 6 mice per treatment group, *P < 0.05, **P < 0.01

To explore whether CSC exosomes affect CCRCC in vivo, 5 mg CSC exosomes or control exosomes were injected into nude mice via tail vein once every 3 days for 2 week. Meanwhile, ACHN and 786-O cells were implanted subcutaneously in the flank of nude mice, or injected into tail veins of nude mice. Our results demonstrated that CSC exosomes accelerated the growth of tumors. The average tumor size and weight in the CSC exosome groups were significantly greater than those in the control group (Fig. 5a-c).

Four weeks following injection of ACHN and 786-O cells transduced to express luciferase, mice were injected intraperitoneally with luciferin and imaged using a Xenogen IVIS imaging system. Tumors were present in 100% of mice, and CSC exosomes promoted the CCRCC cells lung metastasis (Fig. 5d). Thus, above data indicated that CSC exosomes accelerates CCRCC cells proliferation and lung metastasis in vivo. It was noted that M-S-Exo elicited greater effects than S-Exo on proliferation and lung metastases.

### Role of CD103 in directing metastatic sites of exosomes

**Fig. 6:**
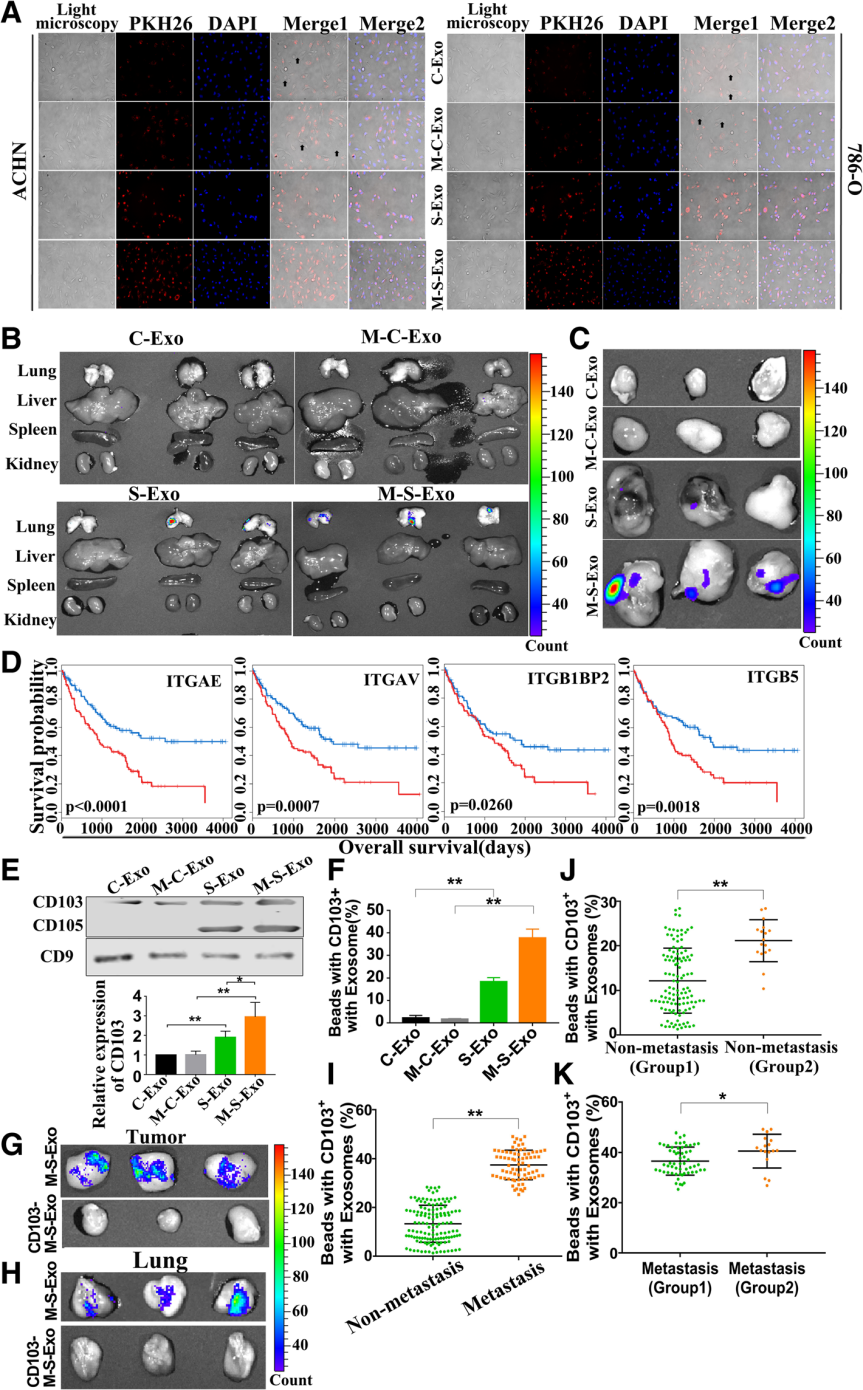
CD103^+^ CSCs exosomes contribute to the cancer cell organotropism. **a** Relative ability of varying types of exosomes to fuse with CCRCC cells, as assessed by fluorescent labelling with PKH26 membrane dye in ACHN and 786-O cells. Arrows indicate no fusion. **b** Comparative distributions of PKH26-labeled exosomes of different types in the main organs of nude mice. *n* = 6 mice per treatment group. **c** Comparative distributions of PKH26-labeled exosomes of varying types in the resected tumor xenografts. *n* = 6 mice per treatment group. **d** Correlation of expression levels of four integrins (ITGAE/CD103, ITGAV, ITGB1BP2, and ITGB5) with the overall survival rate of CCRCC patients. **e** The CD103 protein levels in C-Exo, M-C-Exo, S-Exo and M-C-Exo. *n* = 5, *P < 0.05, **P < 0.01 relative to the controls. **f** The ratio of CD103^+^ exosomes over total exosomes of four different types determined by flow cytometry quantification. **g** & **h** Removal of CD103^+^ exosomes (CD103^−^ M-S-Exo) rendered a loss of the tumorigenesis and aggregation of M-S-Exo in tumor and lung. *n* = 6 mice per treatment group. **i** The ratio of CD103^+^ exosomes over total exosomes in the blood samples from non-metastatic and metastatic CCRCC patients, determined by flow cytometry quantification. **j** & **k** Before and after cancer metastasis in 17 CCRCC patients, the ratio of CD103^+^ exosomes in the blood samples were detected and compared with other patients without and with metastasis respectively. Group 2 represents the exosomes isolated from 17 CCRCC patients

We have shown in the above subsections that among the exosomes of different sources or origins, M-S-Exo demonstrated the strongest cancerogenic potential compared with S-Exo, C-Exo and M-C-Exo. We wanted to understand what mechanisms underlie such a difference. To this end, we compared the cell fusion capacity and tissue/organ targeting of exosomes under both in vitro and in vivo conditions. The ability of exosomes to fuse with ACHN and 786-O cells was detected 4 h after treatment with labeled exosomes. As depicted in Fig. 6a, S-Exo and M-S-Exo were both able to fuse with both ACHN and 786-O cells. Comparing with cancer exosomes, all CCRCC cells were fused with, and M-S-Exo present more efficiency. Moreover, S-Exo and M-S-Exo were densely aggregated in lung, whereas C-Exo and M-C-Exo were only minimally presented in these tissues (Fig. 6b). CCRCC cells were injected into the flank of nude mice. Meanwhile, exosomes were injected into tail veins of mice once every 3 days for 2 weeks. After 4 weeks of cells injection, labeled exosomes were last administrated into mice, then tumors were collected and the fluorescence value was detected at 12 h after labeled exosome administration (R-Fig. 6c). Our results showed that S-Exo and M-S-Exo were densely aggregated in tumor and M-S-Exo had stronger ability to target tumor.

Hoshino et al. revealed that tumour exosome integrins determine organotropic metastasis [17]. We therefore subsequently analyzed the integrins in CCRCC patients utilizing the mRNA expression profiles from The Cancer Genome Atlas (TCGA). Thirteen integrin mRNAs were found overexpressed in CCRCC patients at stages III and IV relative to the subjects at stages I and II, including four mRNAs that are believed to be the negative factor to survival (ITGAE, ITGAV, ITGB1BP2 and ITGB5) (Fig. 6d). Our results showed that only CD103/*ITGAE* was overexpressed in CSC exosomes, and the protein levels of CD103 were significantly higher with M-S-Exo than with S-Exo (Fig. 6e). Furthermore, the flow cytometry results indicated that M-S-Exo contained a higher ratio of CD103^+^ exosomes (Fig. 6f). To verify the role of CD103 in guiding exosomes to their destination, CD103^+^ exosomes were removed from total M-S-Exo, and the labeled M-S-Exo and CD103^−^ M-S-Exo were then injected to mice, respectively. Our data demonstrated that the CD103^+^ exosomes-deprived M-S-Exo lost their ability to target tumor and lung, as indicated by abrogation of aggregation of M-S-Exo in tumor and lung after CD103^+^ exosomes had been removed (Fig. 6g & h).

Finally, blood samples of CCRCC patients with (Additional file 1: Table S1) (76) or without (133) metastatic carcinoma were collected and analyzed using flow cytometry for the count CD103^+^ exosomes. Our results showed that the ratio of CD103^+^ exosomes over total exsocomes was increased in patients with metastatic carcinoma (Fig. 6i). Of the 133 CCRCC patients, 17 of them had metastasis and died of metastasis within 3 years after surgery. Then, we analyzed the relative ratio of CD103^+^ exosomes of these 17 patients. We found that the ratio of CD103^+^ exosomes in these 17 patients was present higher level than the other 116 patients without metastasis (Fig. 6j). Moreover, blood samples were detected when the 17 patients present metastasis at the time of diagnosis. It was indicated that the ratio of CD103^+^ exosomes in the 17 patients was increased compared with patients with other metastatic carcinoma (Fig. 6k).

## Discussion

It was reported up to 30% of all renal cell carcinomas have distant metastases at the time of diagnosis. Lung metastases in renal cell carcinoma is the most common among various sites, accounting for 52% of the total [1–3]. More frustratingly, CCRCC patients with metastasis are facing with rather limited therapeutic approaches in the clinic at present. Therefore, it is necessary to uncover the intertwined mechanisms behind of metastatic initiation and occurrence of CCRCC and identify efficient therapeutic targets for metastatic CCRCC. In this study, we collected the CSC and cancer exosomes respectively derived from metastatic and non-metastatic CCRCC patients and investigated their relative strengths in conferring the malignancy to tumors. The main findings of the present study can be summarized as following. (1) CSC exosomes were significantly more malignant than cancer exosomes. (2) CSC exosomes strongly promoted EMT thereby the migration and invasion capacities. (3) MiR-19b-3p incorporated into CSC exosomes and transferred by CSC exosomes to cancer cells played the key role in EMT via targeting PTEN. (4) An integrin CD103 enriched in CSC exosomes was a critical determinant of organotropic metastasis of CSC exosomes thereby miR-9b-3p. The larger proportion of CD103^+^ exosomes over total exosomes in CSCs of metastatic patients seemed to be a crucial factor in directing metastatic sites of exosomes (Additional file 2: Figure S1). Present reports proved that cancer cell population can obtain some properties of CSCs during the EMT process [30, 31]. In our study, CCRCC cells obtained high ability of metastasis via EMT promotion induced by CSCs-derived exosomes, which similarly act as CSCs.

CSCs play a key role in tumorigenesis and progression of tumors, and published studies have unraveled the evolving process from CSCs to cancer cells [4]. It is believed that CSCs to drive tumor relapse by re-initiating and repopulating new tumors as a cellular and molecular mechanism for the metastasis of cancer [4, 32]. However, there is still little compelling evidence for CSCs to cause metastasis of cancers via exosomes. The present study shed light on the molecular mechanisms for CCRCC metastasis based on exosomes secreted by CSCs. We found that miR-19b-3p enriched in CSC exosomes relative to cancer exosomes mediated the metastatic capacity of CCRCC. Consistently, previous studies have demonstrated that miR-19 family promotes proliferation, invasion, and metastasis of colorectal and breast cancer [33, 34]. It has also been reported that miR-19b mediates EMT in lung cancer [35]. Our results demonstrated that CSC exosomes accelerated EMT via transmitting miR-19b-3p to recipient or target cells. Such malignant effects of miR-19b-3p could be reversed by its antisense inhibitor to knockdown this miRNA.

Exosomes, as a widespread carrier cells, load a variety of cargo including protein, lipids, DNAs, mRNAs, in addition to miRNAs, and function in intercellular communications between cancer cells and their microenvironment through horizontal transfer of information [11, 36]. Studies have highlighted the epigenetic changes such as resistance to cytotoxic drug and angiogenic properties conferred by exosomes in the recipient cells through this cargo-delivery process [37]. It is widely believed that the tumor-derived exosomes with distinct integrin expression patterns up-taken by organ-specific cells prepare the pre-metastatic niche. Lyden et al found that exosomes from highly metastatic melanoma cells develops more metastatic lesions by permanently ‘educating’ bone marrow progenitors through the receptor tyrosine kinase MET [21, 38]. Exosomes serve as mediators to relay bystander effects of secreting CSCs into recipient cells for priming a tumor permissive environment and played a role in tumor progression [39]. Likewise, we found CSC exosomes from metastatic CCRCC patients cells promoted tumor growth, and gathered in cancer cells and lung with the greatest efficacies among various exosomes (CSC exosomes from non-metastatic CCRC patients, cancer exosomes from metastatic and non-metastatic CCRCC patients). To explain the results, we delineated for the first time that CD103^+^ CSC exosomes possess much stronger organotropic property than CD103^−^ CSC exosomes. Our exosome proteomics analysis and bio-informatics database mining showed that only CD103/*ITGAE* was enriched in CSC exosomes. More importantly, we verified the circulating CD103^+^ exosomes as a biomarker for the progression and metastasis of CCRCC.

In conclusion, our findings suggest that CSC exosomes promotes EMT of CCRCC via transport of miR-19b-3p. Furthermore, M-S-Exo derived from CSCs of metastatic CCRCC patients has stronger fusion and contains a high proportion of CD103^+^ exosomes, which presents a strong EMT regulatory effect. miR-19b-3p in CSC exosomes might be considered a therapeutic target to the development of new approaches for preventing metastasis of CCRCC. In addition, the pivotal effect of CD103 in guiding organotropism suggest that depleting CD103^+^ CSC exosomes might represent another novel strategy for the treatment of metastatic CCRCC patients.

## Additional files

Additional file 1: **Table S1.** Patient and Tumor Characteristics. (DOC 39 kb)

Additional file 2: **Figure S1.** Schematic diagram. (TIF 1191 kb)

## Abbreviations

3′-UTR: 3′-Untranslated region
anti-miR: Antisense inhibitor of miRNA
CCRCC: Clear cell renal cell carcinoma
C-Exo: Cancer cell exosomes isolated from medium of non-metastaticCCRCC patients
CSCs: Cancer stem cells
EMT: Epithelial-mesenchymal transition
M-C-Exo: Cancer cell exosomes isolated from medium of metastaticCCRCC patients
miRNA: MicroRNA
M-S-Exo: CSC exosomes isolated from medium of metastatic CCRCC patients
qRT-PCR: Quantitative real-time polymerase chain reaction
S-Exo: CSC exosomes isolated from medium of non-metastatic CCRCC patients
