# Correction to: CD103-positive CSC exosome promotes EMT of clear cell renal cell carcinoma: role of remote MiR-19b-3p

**DOI:** 10.1186/s12943-020-01261-y

**Published:** 2020-09-24

**Authors:** Lu Wang, Guang Yang, Danfeng Zhao, Jiaqi Wang, Yang Bai, Qiang Peng, Hongzhi Wang, Ruizhe Fang, Guang Chen, Zhichao Wang, Keliang Wang, Guangbin Li, Yinhui Yang, Ziqi Wang, Pengyu Guo, Li Peng, Dayong Hou, Wanhai Xu

**Affiliations:** 1 Department of Urology (Heilongjiang Key Laboratory of Scientific Research in Urology), the Fourth Hospital of Harbin Medical University, No. 37 Yi-Yuan Street, Nangang District, Harbin, Heilongjiang Province 150081 People’s Republic of China; 2 Department of Neurosurgery, The First Affiliated Hospital of Harbin Medical University, Harbin, Heilongjiang Province People’s Republic of China

**Correction to: Mol Cancer 18, 86 (2019)**

**https://doi.org/10.1186/s12943-019-0997-z**

After the publication of this work [1], the authors reported errors in Fig. 4a and b. The image of “ACHN cells/ NC-48h” in Fig. 4a was unintentionally covered during figure processing, so the image of “ACHN cells/ C-Exo 48h” in Fig. 2b was showed duplicately. In Fig. 4b, the images of “786-O cells” were accidentally deleted, but the images of “ACHN cells” were duplicative displayed. The errors do not change the original conclusions of the article. The authors apologize for any inconvenience that the inaccuracy may have caused.


