# Supplementary material for: CD103-positive CSC exosome promotes EMT of clear cell renal cell carcinoma: role of remote MiR-19b-3p
Source: Mol Cancer. 2019 Apr 11;18:86. doi: 10.1186/s12943-019-0997-z (PMC6458839; doi:10.1186/s12943-019-0997-z)
Supplement: Supplementary file 1 — Table S1. Patient and Tumor Characteristics. (DOC 39 kb) [file 12943_2019_997_MOESM1_ESM.doc]

Additional file 1: Table S1. Patient and Tumor Characteristics

| **Variables** | **No. of Patients (%)** |
| --- | --- |
|
| **Sex** |  |
| Female | 66（34.38%） |
| Male | 126（65.63%） |
| **Age (y)** |  |
| ≤60 | 111（57.81%） |
| >60 | 81（42.19%） |
| **Tumor size** (cm) |  |
| ≤4 | 79（41.15%） |
| >4 | 113（58.85%） |
| **Location** |  |
| Left | 86（44.79%） |
| Right | 106（55.21%） |
| **Histological** |  |
| I, II | 133（69.27%） |
| III, IV | 59（30.73%） |
