# Supplementary figures and images for: CD103-positive CSC exosome promotes EMT of clear cell renal cell carcinoma: role of remote MiR-19b-3p
Source: Mol Cancer. 2019 Apr 11;18:86. doi: 10.1186/s12943-019-0997-z (PMC6458839; doi:10.1186/s12943-019-0997-z)

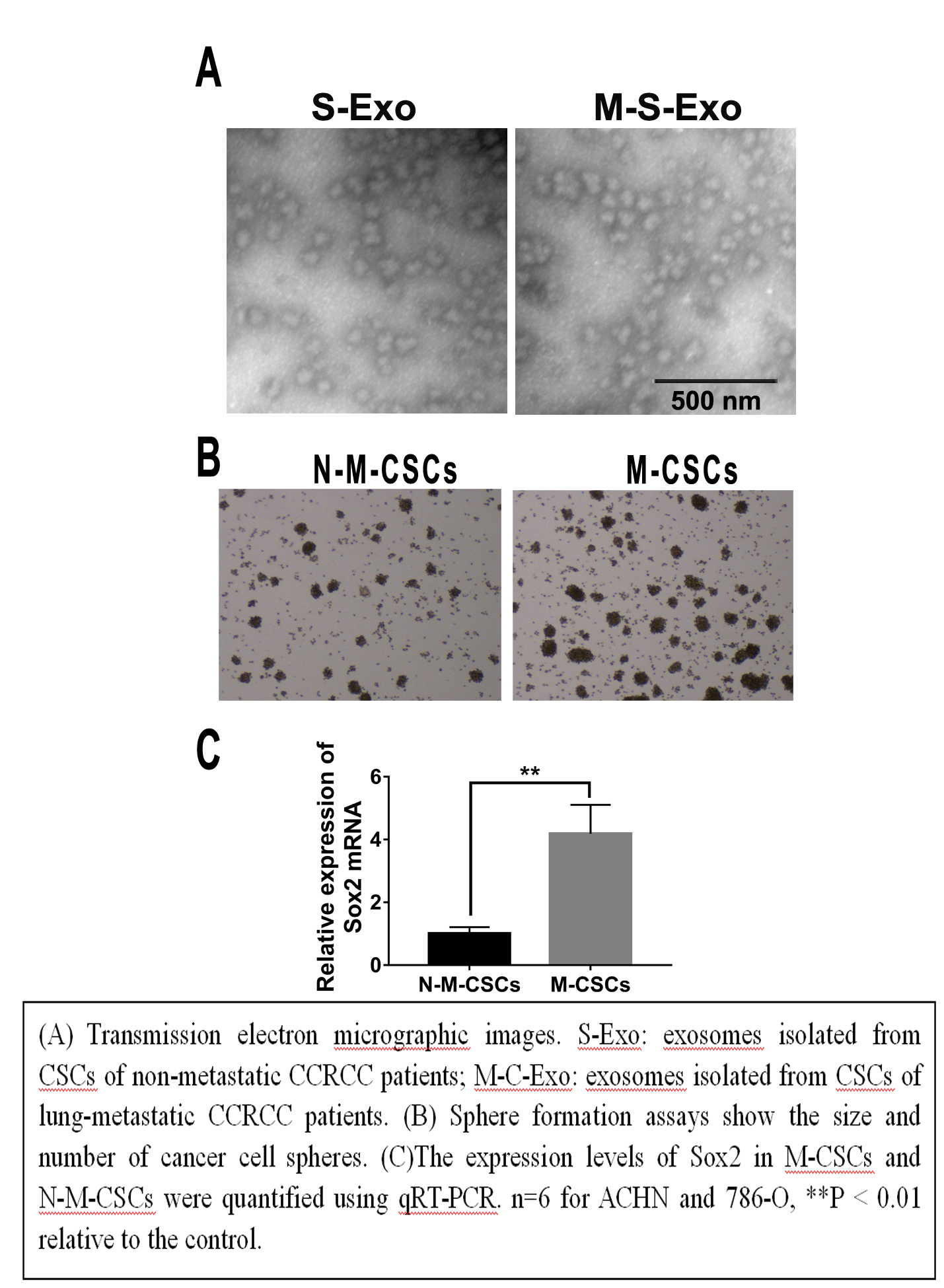

Supplement: Supplementary file 2 — Figure S1. Schematic diagram. (TIF 1191 kb) [file 12943_2019_997_MOESM2_ESM.tif]
